# Analysis of reference genes stability and histidine kinase expression under cold stress in *Cordyceps militaris*

**DOI:** 10.1371/journal.pone.0236898

**Published:** 2020-08-12

**Authors:** Yong-Nan Liu, Bi-Yang Liu, You-Chu Ma, Hai-Long Yang, Gao-Qiang Liu

**Affiliations:** 1 Hunan Provincial Key Laboratory for Forestry Biotechnology & International Cooperation Base of Science and Technology Innovation on Forest Resource Biotechnology, Central South University of Forestry and Technology, Changsha, China; 2 College of Environmental & Life Science, Wenzhou University, Wenzhou, China; ICAR-Directorate of Mushroom Research, INDIA

## Abstract

The development of fungal fruiting bodies from a hyphal thallus is inducible under low temperature (cold stress). The molecular mechanism has been subject to surprisingly few studies. Analysis of gene expression level has become an important means to study gene function and its regulation mechanism. But identification of reference genes (RGs) stability under cold stress have not been reported in famous medicinal mushroom-forming fungi *Cordyceps militaris*. Herein, 12 candidate RGs had been systematically validated under cold stress in *C*. *militaris*. Three different algorithms, geNorm, NormFinder and BestKeeper were applied to evaluate the expression stability of the RGs. Our results showed that *UBC* and *UBQ* were the most stable RGs for cold treatments in short and long periods, respectively. 2 RGs (*UBC* and *PP2A*) and 3 RGs (*UBQ*, *TUB* and *CYP*) were the suitable RGs for cold treatments in short and long periods, respectively. Moreover, target genes, two-component-system histidine kinase genes, were selected to validate the most and least stable RGs under cold treatment, which indicated that use of unstable expressed genes as RGs leads to biased results. Our results provide a good starting point for accurate reverse transcriptase quantitative polymerase chain reaction normalization by using *UBC* and *UBQ* in *C*. *militaris* under cold stress and better support for understanding the mechanism of response to cold stress and fruiting body formation in *C*. *militaris* and other mushroom-forming fungi in future research.

## Introduction

*Cordyceps militaris*, a famous traditional Chinese medicine, has been used as a healthy food for a long time in China. The *C*. *militaris* fruiting body has anti-inflammatory [1], anti-tumor [2], anti-influenza virus [3] and radio-protection [4] functions. Methods for commercial production of fruiting bodies of this fungus have been established in artificial media [5, 6] or with insects, such as silkworm *Bombyx mori* pupae [7]. But, the molecular mechanism of fruiting body formation is not well understood. The essential role of light in fruiting body development in *C*. *militaris* was demonstrated in previous study [8]. Inactivation of a blue-light receptor gene *white collar-1* (*wc-1*) resulted in thicker aerial hyphae, disordered development in *C*. *militaris* [9]. Further study showed that the fruiting body primordia could form in the Drosophila, Arabidopsis, Synechocystis and Human (DASH) type cryptochromes (CRYs) gene mutant strains, but the fruiting bodies were unable to elongate normally, and *cry-DASH* and *Cmwc-1* exhibited interdependent expression under light in *C*. *militaris* [10].

Compared to light, temperature is another pivotal factor influencing fruiting body formation from a hyphal thallus which represents a transition from simple to complex multicellularity [11]. The fruiting body formation of various mushrooms depends on low temperature (cold stress) induction. For example, the fruiting body development was induced by shifting the cultivation condition (25°C for mycelia) to lower temperature (15°C) in *Flammulina velutipes* [12]. In *Pleurotus ostreatus* the mycelia were cultured at 20°C for 30 days in a sawdust-medium, and then the temperature was lowered to 15°C to induce fruiting body development [13]. In *C*. *militaris*, after the mycelia were cultured under static conditions at 25°C on potato dextrose broth medium for 10 days, the mycelia were injected into pupae and then the inoculated pupae were kept at 20°C to induce the fruiting bodies [7]. However, the molecular mechanism of mycelial development to fruiting body under cold induction has been subject to surprisingly few studies, which will seriously restrict the further development of commercial production of mushrooms fruiting bodies.

Two-component signal transduction is commonly used by eubacteria, archea, and eukaryotes as a stimulus–response coupling mechanism to sense and respond to changes in many different environmental conditions, especially temperature sensing [14]. They consist of a histidine kinase (HK) and a response regulator. The histidine kinase DesK from *Bacillus subtilis* is mechanistically the best understood. After a temperature downshift DesK gets auto-phosphorylated at the conserved histidine residue and donates the phosphate group to the conserved aspartate residue of DesR. The phosphorylated DesR activates the transcription of the *des* gene [15]. However, little is known the role of *HK* genes in cold stress response and fruiting body formation in *C*. *militaris* and other mushroom-forming fungi.

Due to its high sensitivity, specificity, and reliable quantification, real-time quantitative polymerase chain reaction (qRT-PCR) is an important tool to understand the complex signaling networks when an organism is submitted to different stimuli [16, 17]. However, the results of qRT-PCR are often inaccurate because of the occurrence of errors in the primer specificity, complementary DNA (cDNA) synthesis and PCR amplification [18]. Therefore, qRT-PCR must be normalized using reference genes that show a stable expression. However, validation of suitable RGs for expression analysis under cold stress have not been conducted in *C*. *militaris* and many other mushroom-forming fungi. In this study, we systematically identified 12 candidate RGs in *C*. *militaris* that were measured using qRT-PCR. Three different algorithms, geNorm, NormFinder and BestKeeper were applied to evaluate the expression stability of the 12 candidate RGs under cold treatment in *C*. *militaris*. Moreover, the relative expression levels of two-component-system HK genes were analyzed under cold stress conditions with three different normalization strategies. The RGs screened out in this paper could provide robustness to study the regulatory mechanism of cold induced fruiting body formation from mycelia in *C*. *militaris* and other mushroom-forming fungi.

## Materials and methods

### Strains and culture conditions

A laboratory and commercial strain of *C*. *militaris* (CGMCC 3.14242) from China General Microbiological Culture Collection Center was used. The *C*. *militaris* was cultured at 25°C in dark with an artificial medium containing 20 g rice, 0.5 g powder of silkworm pupae and 25-mL nutrient solution (glucose 20 g, KH_2_PO_4_ 2 g, MgSO_4_ 1 g, ammonium citrate 1 g, peptone 5 g, vitamin B_1_ 20 mg, and 1,000-mL distilled water).

### Cold stress

After cultured for 25 days at 25°C in dark, the *C*. *militaris* mycelia were transferred to 20°C in dark for different treatments under cold stress for different time periods including short periods (0-, 0.5-, 1-, 2-, 4- and 8-hours) and long periods (0-, 1-, 2-, 4- and 8-d). After treatment, mycelia samples were immediately frozen in liquid nitrogen and stored at −80°C in a deep freezer until RNA isolation. Untreated mycelia samples were used as the control. Each treatment was repeated for 3 times.

### RNA isolation and cDNA synthesis

RNA isolation and cDNA synthesis were performed as previously described method [19]. Briefly, 0.2 g mycelia were collected and subsequently were disrupted under liquid nitrogen conditions. The RNA Isolation Kit (TaKaRa, China) was used to extract total RNA, treated with DNase I to avoid genomic DNA contamination. 2 μg total RNA of each sample was added to 20 μL reverse transcription reaction system to synthesize cDNA. The cDNA was subjected to 10-fold serial dilution for determining the amplification efficiency of qRT-PCR upon cold treatments.

### Real-time quantitative polymerase chain reaction conditions

qRT-PCR experiments were performed using a previously described method [20]. Briefly, all genes were amplified by initial heating at 95°C for 10 min, followed by 40 cycles of 95°C for 15 s, 58°C for 15 s, and 68°C for 15 s. At the final amplification cycle, the specificity of PCR reactions was checked through the use of melting curve analysis (60–95°C in increments of 0.5°C every 5 s). Negative controls were included to ensure the suitability of the assay conditions. Each experiment described above was repeated independently at least for 3 times.

### Selection of candidate reference genes

In this study, 12 genes (namely *ACT*, *TUB*, *UBC*, *EF-1α*, *GAPDH*, *PP2A*, *UBQ*, *PGK*, *RPS*, *FBOX*, *CYP* and *GTPB*) were selected as candidate RGs. Our gene panel contained (1) traditional RGs such as tubulin (*TUB*), actin (*ACT)*, polyubiquitin (*UBQ*), glyceraldehyde-3-phosphate dehydrogenase (*GAPDH*) and translation elongation factor-1*α* (*EF-1α*); (2) genes chosen on the basis of stable expression in several RNA sequencing and quantitative PCR experiments, such as cyclophilin (*CYP*) [21] and ubiquitin-conjugating enzyme (*UBC*) [22]; and (3) novel stable RGs in cold stress such as phosphoglycerate kinase (*PGK*) and serine/threonine protein phosphatase 2A (*PP2A*) [23]. Details of these genes are provided in Table 1. These genes were annotated with a *C*. *militaris* genome database (National Center for Biotechnology Information accession GSE28001 and AEVU00000000) [24] and used to design primers. The specific primers for two-component-system histidine kinase (HK) genes were listed in S1 Table.

**Table 1:** Details on primers used for quantitative reverse transcriptase polymerase chain reaction analysis.

Gene	Annotation	Accession No.	Primer Sequences (5’-3’)	Amplification Size (bp)	Efficiency (%)
*ACT*	Actin cytoskeleton protein	XM_006669552.1	F:CAACAACTTCCTGACGGGC	219	99.6
			R:TCCTTGGGCTTCTGCTGAC		
*TUB*	Tubulin	XM_006669203.1	F:ATGTCGTTCGTCGTGAGG	208	97.5
			R:AGAGTGGCGTTGTAGGGT		
*UBC*	Ubiquitin-conjugating enzyme	XM_006672277.1	F:ACCATTGACACGAGCCAGTT	204	101.3
			R:GCCCATGTAAGCCTCCTCA		
*EF-1α*	Elongation factor 1-alpha	XM_006665968.1	F:TATCGGAACTGTGCCTGT	200	106.2
			R:CGTTACCACGACGGATTT		
*GAPDH*	Glyceraldehyde 3-phosphate dehydrogenase	XM_006669697.1	F:CATCCACTCCTACACTGCTAC	220	102.3
			R:CTCAAGACGAACAGTCAGGT		
*PP2A*	Serine/threonine protein phosphatase 2A	XM_006673033.1	F:CCTCCTACAGTCGTCATCAGC	198	105.7
			R:AGAAATGTCAAAGCGAGA		
*UBQ*	Polyubiquitin	XM_006672469.1	F:TCAAAGAAGATAATGGTAACG	209	102.5
			R:GTATGGGTTCTCGGAAAGGT		
*PGK*	Phosphoglycerate kinase	XM_006673408.1	F:GCTCAAGCCCGTCGTTTC	159	98.9
			R:CCCTCCTCCTCAATGTGG		
*RPS*	Ribosomal protein S25	XM_006665399.1	F:AAGTGGTCTAAGGGCAAGG	182	97.8
			R:TTCTCCTCCAGGTCGGTAA		
*FBOX*	F-box protein	XM_006672810.1	F:CCGATGACAACGACAGCGAC	224	101.3
			R:GTAGTTGACCGTGGAGATGT		
*CYP*	cyclophilin	XM_006674778.1	F:TTTTCCGCCTTATTCCACC	197	95.5
			R:TCCAGAGCATCAAATCCCT		
*GTPB*	GTP-binding protein	XM_006674648.1	F:TAAGAAGCCCAAGAAGAAAA	181	103.4
			R:GTCCCACAGGTTCAGCGT		

F, forward; R, reverse.

### Data analysis to select the internal reference gene

The expression stability of the candidate RGs was analyzed using geNorm [16], NormFinder [25] and BestKeeper [26] as previously described.

### Abbreviations

ACT: actin cytoskeleton protein; cDNA: complementary DNA; Cq: quantification cycle; CV: coefficient of variation; CYP: cyclophilin; EF-1α: elongation factor 1-alpha; FBOX: F-box protein; GAPDH: glyceraldehyde 3-phosphate dehydrogenase; GTPB: GTP-binding protein; HK: histidine kinase; *M* value: measurement value; PGK: phosphoglycerate kinase; PP2A: serine/threonine protein phosphatase 2A; RGs: reference genes; RPS: ribosomal protein S25; qRT-PCR: real-time quantitative polymerase chain reaction; SD: standard deviation; TUB: tubulin; UBC: ubiquitin-conjugating enzyme; UBQ: polyubiquitin; *Vn/Vn+1*: pairwise variation.

## Results

### Expression profiling of 12 candidate reference genes in *C*. *militaris*

**Fig 1:**
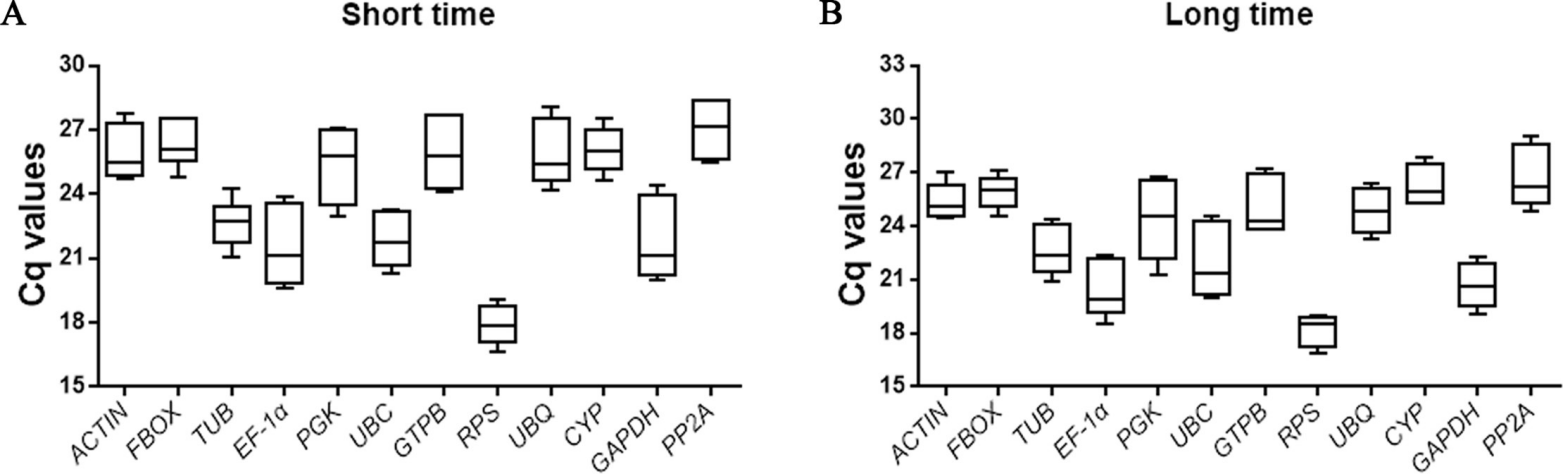
The range of quantification cycle (Cq) values of the 12 candidate reference genes in *C*. *militaris* under cold stress for short (A) and long periods (B).

12 genes (namely *ACT*, *TUB*, *UBC*, *EF-1α*, *GAPDH*, *PP2A*, *UBQ*, *PGK*, *RPS*, *FBOX*, *CYP* and *GTPB*) were selected as candidate RGs to determine the most stable RGs under cold treatments. We first calculated the PCR efficiency of twelve pairs of primers according to the previously reported method [27]. As shown in Table 1, qRT-PCR efficiency of the 12 genes ranged from 95.5% (*CYP*) to 106.2% (*EF-1α*) which fell within the acceptable range [28]. The quantification cycle (Cq) values of the 12 genes exhibited a high variation ranging and shown in Fig 1. The Cq values ranged from 16.6 to 28.4 and 16.9 to 29.0 of short periods (Fig 1A) and long periods (Fig 1B), respectively. *RPS* and *PP2A* was the most and least transcribed, respectively, across all the tested samples (Fig 1A and 1B). Three different algorithms (geNorm, NormFinder and BestKeeper) were used to analyze the expression stability of the 12 genes in the next section.

### geNorm analysis

**Fig 2:**
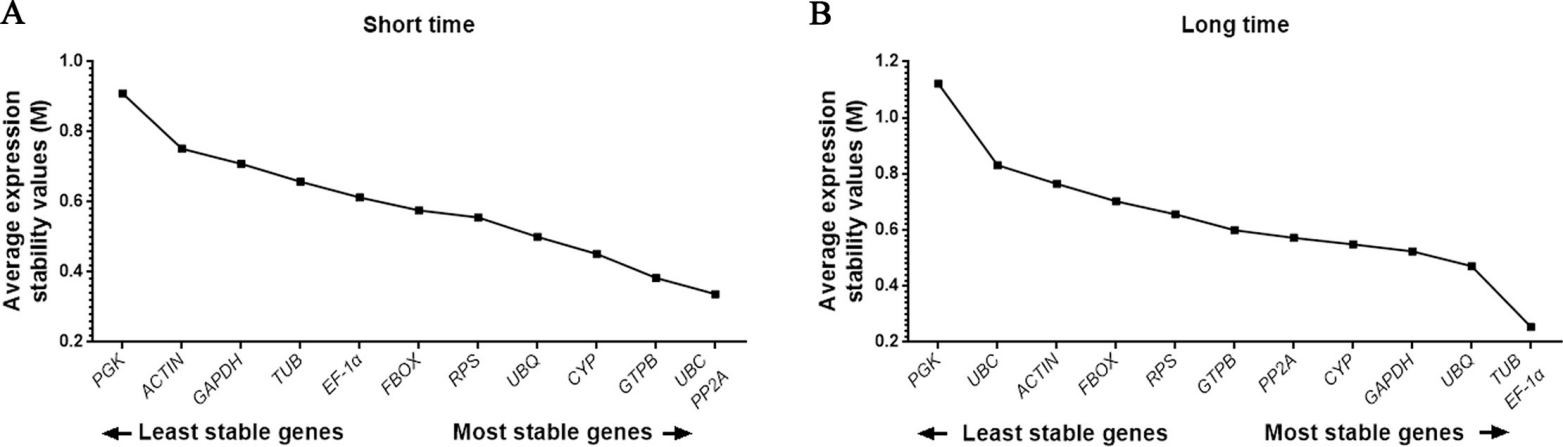
geNorm analysis of the average expression stability values (*M*) of the 12 candidate reference genes under cold stress for short (A) and long periods (B). A higher *M* value indicates more unstable expression.

The geNorm algorithm calculates the expression stability factor (*M*). The *M* value is defined as the average pairwise variation of a particular gene with all other reference genes within a given group of cDNA samples, where a low value represents stable and a high value represents unstable expression. As determined by geNorm (Fig 2), expression stability (*M* values) of the 12 genes both in short and long periods were within the acceptable range (*M* < 1.5) [16]. The *M* value ranged from 0.91 (*PGK*) to 0.34 (*UBC* and *PP2A*) in short periods (Fig 2A) and 1.12 (*PGK*) to 0.25 (*TUB* and *EF-1α*) in long periods (Fig 2B). The genes were ranked from the highest *M* value (least stable) to the lowest *M* value (most stable): *PGK*, *ACT*, *GAPDH*, *TUB*, *EF-1α*, *FBOX*, *RPS*, *UBQ*, *CYP*, *GTPB*, *UBC* and *PP2A* in short periods (Fig 2A), *PGK*, *UBC*, *ACT*, *FBOX*, *RPS*, *GTPB*, *PP2A*, *CYP*, *GAPDH*, *UBQ*, *TUB* and *EF-1α* in long periods (Fig 2B).

### NormFinder analysis

Next, the expression stability of the 12 candidate RGs was analyzed using NormFinder to confirm the geNorm results. In short periods, *UBC* and *UBQ* were the most stable; their stability values were 0.141 and 0.244, respectively (Table 2). In long periods, *CYP* and *UBQ* were the most highly ranked; their stability values were 0.123 and 0.202, respectively (Table 3). Combining the results of geNorm and NormFinder analysis, *UBC* followed by *PP2A*, *GTPB* and *UBQ* were the most stable RGs in short periods (Table 2 Mean Rank); *UBQ* followed by *TUB*, *CYP* and *GAPDH* were the most stable RGs in long periods (Table 3 Mean Rank).

**Table 2:** Gene expression stability under short time cold stress ranked by geNorm, NormFinder and BestKeeper.

Genes	geNorm		NormFinder		Mean Rank^a^	BestKeeper	
	*M* Value	Rank Order	Stability Value	Rank Order		CV	SD
***UBC***	0.336	1.5	0.141	1	1	4.501	0.882
***PP2A***	0.336	1.5	0.248	3	2	3.596	0.872
*GTPB*	0.383	3	0.287	4	3	4.647	1.203
*CYP*	0.451	4	0.303	5	5	2.858	0.744
*UBQ*	0.500	5	0.244	2	3	4.725	1.222
*RPS*	0.555	6	0.409	7	6	3.969	0.710
*FBOX*	0.575	7	0.381	6	6	3.115	0.819
*EF-1α*	0.612	8	0.419	8	8	6.658	1.431
*TUB*	0.657	9	0.561	11	9	3.317	0.751
*GAPDH*	0.708	10	0.541	10	9	7.141	1.557
*ACT*	0.751	11	0.490	9	9	3.924	1.016
*PGK*	0.908	12	1.116	12	12	6.299	1.600

**Table 3:** Gene expression stability under long time cold stress ranked by geNorm, NormFinder and BestKeeper.

Genes	geNorm		NormFinder		Mean Rank^a^	BestKeeper	
	*M* Value	Rank Order	Stability Value	Rank Order		CV	SD
***TUB***	0.254	1.5	0.308	4	2	4.934	0.711
*EF-1α*	0.254	1.5	0.360	6	5	6.359	1.930
***UBQ***	0.470	3	0.202	2	1	4.066	0.701
*GAPDH*	0.523	4	0.247	3	4	4.589	0.949
***CYP***	0.548	5	0.123	1	3	3.560	0.693
*PP2A*	0.571	6	0.404	7	7	5.381	1.442
*GTPB*	0.599	7	0.319	5	6	5.670	1.426
*RPS*	0.655	8	0.489	8	8	3.882	0.704
*FBOX*	0.701	9	0.543	9	9	2.487	0.644
*ACT*	0.764	10	0.641	10	10	2.935	0.743
*UBC*	0.830	11	0.730	11	11	8.127	1.792
*PGK*	1.121	12	1.733	12	12	7.325	1.786

### Optimal number of reference gene for normalization across the experimental sets

**Fig 3:**
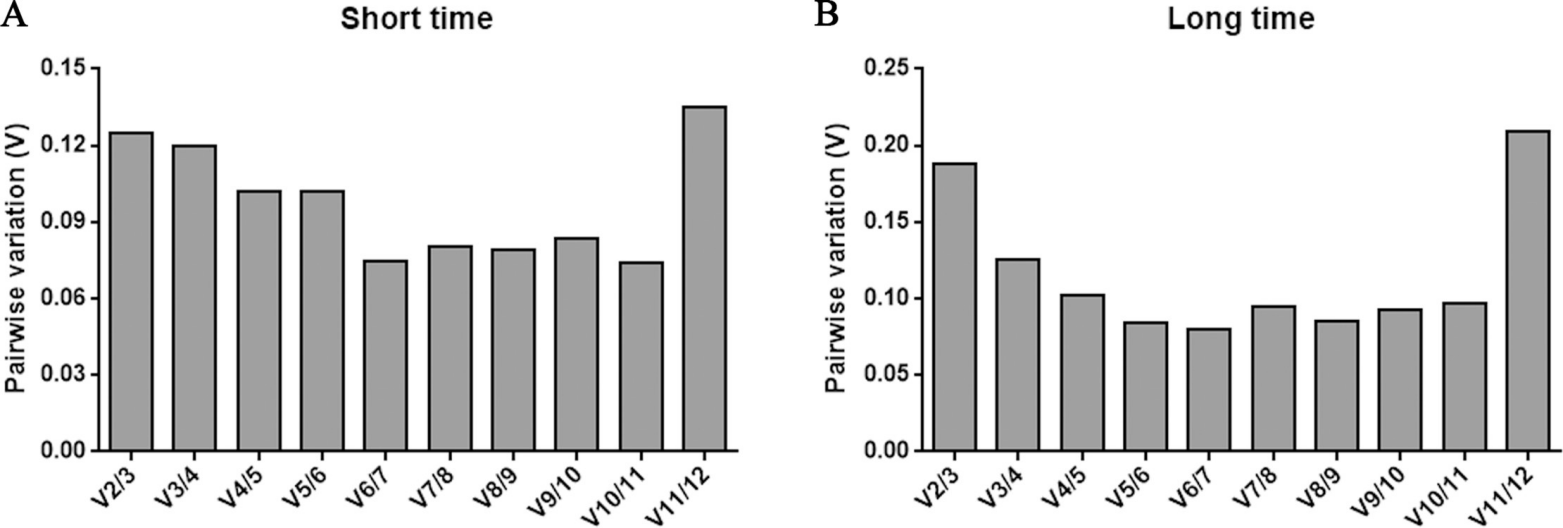
geNorm analysis of the pairwise variation (*V*) of the 12 candidate reference genes under cold stress for short (A) and long periods (B). The pairwise variation *Vn/Vn+1* measured the effect of adding additional reference genes on the normalization factor for cold stress treatment in *C*. *militaris*.

Next, we determine the minimal number of genes for qRT-PCR normalization by estimated pairwise variation (*Vn/Vn+1*) in geNorm. Below 0.15 which was a proposed cut-off *Vn/Vn+1* value, adding an additional RG is not required [16]. According to this principle, the *Vn/Vn+1* value was calculated. In short periods, the *V*2/3 value was 0.124 (< 0.15), therefore, *UBC* together with *PP2A* would be sufficient for purpose (Fig 3A and Table 2). In long periods, compared with *V*2/3 (0.188), *V*3/4 value was 0.125 (< 0.15), suggesting that three RGs, *UBQ*, *TUB* and *CYP* were identified as available for normalization (Fig 3B and Table 3).

### BestKeeper analysis

We further used BestKeeper to analyze the stability of 12 candidate RGs by calculating the standard deviation (SD) and the coefficient of variation (CV) of their Cq values. It has been reported that any studied gene showing a SD in expression lower than 1 can be considered stable, and the most stably expressed gene, exhibiting the lowest CV [26]. As shown in Table 2, in short periods, the top two stable genes *UBC* and *PP2A* with lower CV values 4.501 and 3.596, respectively. And the SD values of *UBC* and *PP2A* were 0.882 (< 1) and 0.872 (< 1), respectively. In long periods, the top three stable genes *UBQ*, *TUB* and *CYP* with lower CV values 4.066, 4.934 and 3.560, respectively. And the SD values of *UBQ*, *TUB* and *CYP* were 0.701 (< 1), 0711 (< 1) and 0.693 (< 1), respectively (Table 3). These BestKeeper results also suggested that *UBC*/*PP2A* and *UBQ*/*TUB/CYP* were stable in short and long periods, respectively.

Taken together, our results suggesting that two RGs *UBC*/*PP2A* and three RGs *UBQ*/*TUB/CYP* were identified as available for qRT-PCR normalization under cold incubation during short and long cold periods, respectively, in *C*. *militaris*.

### Quantitative effects of best and least ranked reference genes on target gene expression

**Fig 4:**
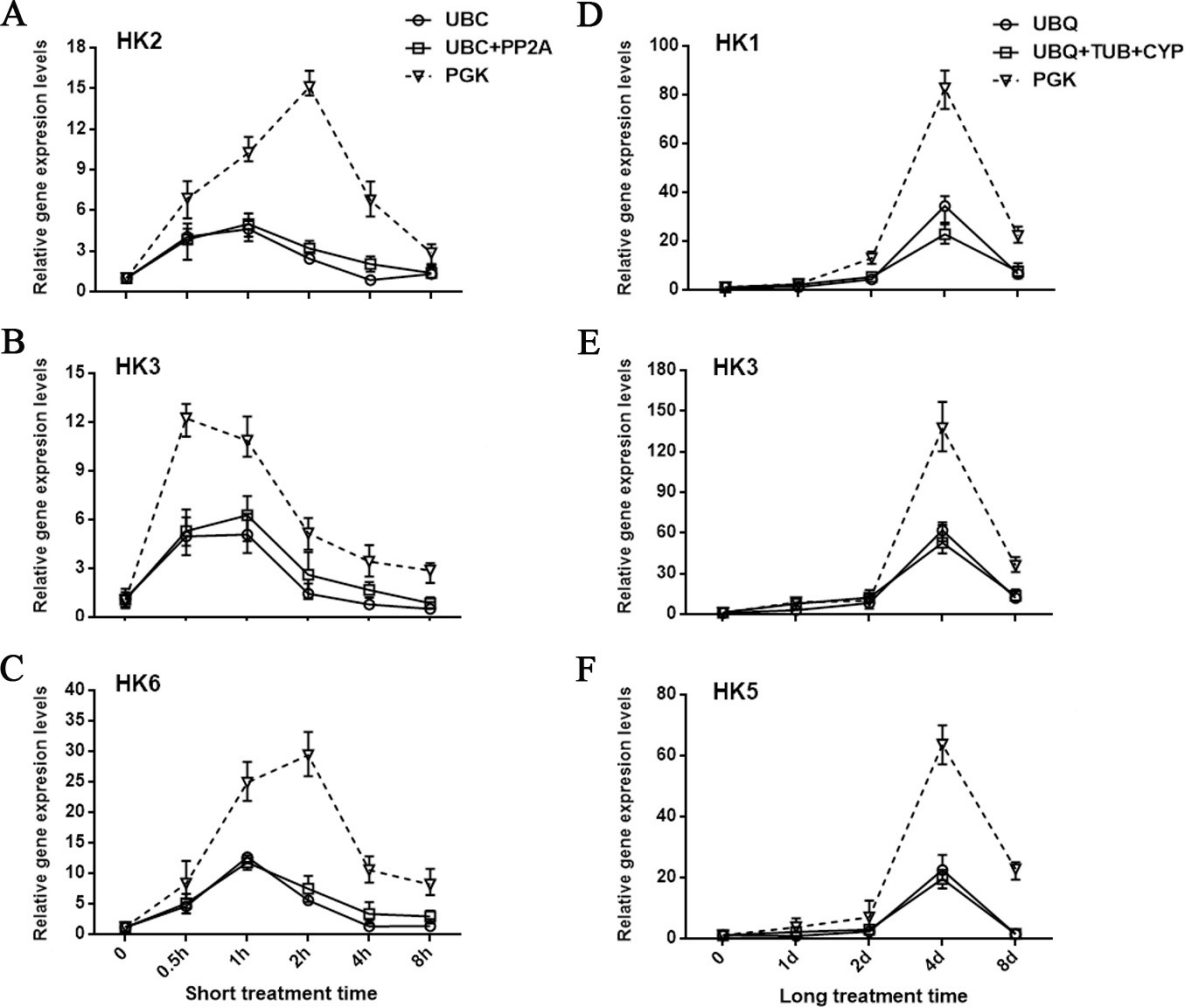
Relative expression levels of HK genes during cold stress conditions using the least stable gene, best ranked gene, or multiple stable reference genes for normalization. The least stable reference gene *PGK*, best ranked reference gene *UBC*, and multiple reference genes *UBC*/*PP2A* were used for normalization to analysis of *HK2* (A) *HK3* (B) and *HK6* (C) expression levels in short periods. The least stable reference gene *PGK*, best ranked reference gene *UBQ*, and multiple reference genes *UBQ*/*TUB*/*CYP* were used for normalization to analysis of *HK1* (D) *HK3* (E) and *HK5* (F) expression levels in long periods. The average CT value of multiple reference genes is used to analysis of *HK* gene expression levels in the strategy of multiple reference genes were used for normalization.

To validate the utility of stable RGs on gene expression analysis, three different strategies, the least stable RG (*PGK*), the best ranked RG and multiple stable RGs were selected to normalize the expression of target gene in short and long periods treatments, respectively, using *PGK*, *UBC* and *UBC*/*PP2A* for short periods RG and P*GK*, *UBQ* and *UBQ/TUB/CYP* for long periods RG. *HK* genes in cold stress response were used as target genes. Based on the annotation with *C*. *militaris* genome database (National Center for Biotechnology Information accession GSE28001 and AEVU00000000), 9 genes (*CmHK1-9*, S1 Table) were identified as *HK* homologous genes. Normalization of the 9 *HK* genes using the three different strategies showed that a significant increase in transcription of *HK2*/*HK3*/*HK6* and *HK1*/*HK3*/ *HK5* were observed under cold incubation during short and long cold periods, respectively (Fig 4). The gene expression levels of other *HK* genes were not shown. When using the best ranked *UBC* or the multiple stable *UBC*/*PP2A* as the RGs in short periods, the upregulated trends for *HK2*, *HK3* and *HK6* genes were consistent and the peak points were observed at 1 h (Fig 4A–4C). When using the best ranked *UBQ* or the multiple stable *UBQ*/*TUB*/*CYP* as the RGs in long periods, the upregulated trends for *HK1*, *HK3* and *HK5* genes were consistent and the peak points were observed at 4 d (Fig 4D–4F), respectively. However, using the least ranked *PGK* as the RG in short periods, the peak points were observed at 2, 0.5, 2 h of *HK2*, *HK3*, *HK6*, respectively (Fig 4A–4C). In addition, when *PGK* was applied as the RG in our experiment, the transcription patterns of *HK* genes were higher than those using the best ranked RG and multiple stable RGs (Fig 4A–4F), which indicated that normalization using the least stable *PGK* as RG resulted in an overestimated relative expression level of the target genes. Thus, our experimental results suggest that it is feasible to use any one or more of *UBC*/*PP2A* and *UBQ*/*TUB*/*CYP* genes as the normalization gene under cold incubation during short and long cold periods, respectively, with *C*. *militaris*.

## Discussion

Compared with microarray and RNA sequencing, qRT-PCR technique has the advantages of high accuracy, high sensitivity, good repeatability and low cost for quantifying gene expression [16, 17]. To ensure the accuracy of qRT-PCR results, it is necessary to analyze the stability of internal RG under a specific experimental condition. A previous study in *C*. *militaris* showed that polymerase II large subunit (*rpb1*) gene was the best RG during all developmental stages examined, while the most common reference genes, *actin*, and *tub*, were not suitable internal controls [29]. In addition, the *actin* also was not suitable RGs under developmental stage, strain, and nutrient source in *Lentinula edodes* [30]. However, suitable RGs for expression analysis under cold stress have not been reported in *C*. *militaris*. In this study, the expression stability of 12 candidate RGs in *C*. *militaris* was systematically analyzed by geNorm, NormFinder and Bestkeeper under the cold treatment. Our results showed that *UBC* and *UBQ* were identified as the most suitable for qRT-PCR normalization under cold incubation during short and long cold periods, respectively. While the *ACT*, a traditionally RG, was not suitable cold stress in *C*. *militaris*. Therefore, we suggest to use two or more RGs (*rpb1*, *UBC* and *UBQ*) to study the expression levels of the fruiting body developmental genes in *C*. *militaris* in the follow-up study.

A large number of studies have been conducted to identify the suitable RGs under cold treatment conditions among different species, but the most stable RGs are diverse (Table 4). In Pineapple (*Ananas comosus* L.), *PP2A* and *CYP* were the stable RGs under cold stress [23]; and in Hulless barley (*Hordeum vulgare* L. var. *nudum*. hook. f.), *PGK* was not stable RG under cold stress [31], which were consistent with our results. However, in *Hemarthria compressa* and *H*. *altissima* leaf tissue, *EF-1α* was the most stable gene under cold stress [32]; and in *Atropa belladonna*, *PGK* was a reliable gene for normalizing gene expression under cold stress conditions [33], which were not consistent with our results. In entomopathogenic fungus *Beauveria bassiana*, both *ACT* and *CYP* were the most stably expressed gene sets under a variety of stress conditions (including cold) [34]. However, in our study, *ACT* was not stable in our experimental conditions. In another mushroom-forming Ascomycota, True morels (*Morchella* spp.), two candidate internal control genes *ACT* and *GAPDH* were not reliable gene for normalizing gene expression under cold stress conditions [35], which was similar to our findings. An interesting result is that, in *Arabidopsis pumila*, both *UBC* and *GAPDH* under cold stress were the most stable RGs [36] (Table 4). But, in our result, *UBC* and *GAPDH* were stable and not stable RGs in short periods, respectively. The similar results were also found in Siberian Wild Rye (*Elymus sibiricus*) that *PP2A* and *ACT* presented the highest degree of expression stability for cold stress [37]. But, in our result, only *PP2A* was stable RG while *ACT* was found to be unstable. The more interesting result is that, in our study, *UBC* was the stable RG in short periods but not the stable RG in long periods. All of these results indicate that the expression stability of the same gene differs in different species. Moreover, the expression stability of the same gene is also not the same under the same treatment at different times.

**Table 4:** The screening results of candidate reference genes of different species under cold stress.

Gene	Species	Expression Stability (Stable/Unstable)	Ref.	Expression Stability In This Study
*PP2A*	*Ananas comosus*	Stable	Chen et al. [23]	Stable (Short periods)
	*Elymus sibiricus*	Stable	Zhang et al. [37]	
*CYP*	*Ananas comosus*	Stable	Chen et al. [23]	Stable (Long periods)
	*Beauveria bassiana*	Stable	Zhou et al. [34]	
PGK	Hordeum vulgare	Unstable	Cai et al. [31]	Unstable (Short and Long periods)
	Atropa belladonna	Stable	Li et al. [33]	
EF-1α	Hemarthria compressa and H. altissima	Stable	Lin et al. [32]	Unstable (Short periods)
*ACT*	*Morchella* spp.	Unstable	Zhang et al. [35]	Unstable (Short and Long periods)
	*Beauveria bassiana*	Stable	Zhou et al. [34]	
	*Elymus sibiricus*	Stable	Zhang et al. [37]	
*GAPDH*	*Morchella* spp	Unstable	Zhang et al. [35]	Unstable (Short and Long periods)
	*Arabidopsis pumila*	Stable	Jin et al. [36]	
*UBC*	*Arabidopsis pumila*	Stable	Jin et al. [36]	Stable (Short periods) Unstable (Long periods)

In *Arabidopsis thaliana*, Arabidopsis histidine kinase 2 (AHK2), AHK3, and cold-inducible type A Arabidopsis response regulators (ARRs) play roles in cold signaling [38]. In addition, stress-sensing in fungi also depends on a signaling cascade comprised of a two-component phosphorylation relay plus a subsequent MAP kinase cascade to trigger gene expression [39]. Moreover, two-component pathways are important determinants of pathogenicity in animal pathogens, such as *Candida albicans* [40], *Cryptococcus neoformans* [41], and plant pathogens including *Fusarium oxysporum* (tomato) [42], *Monilinia fructicola* (brown rot of stone fruit) [43] and *Botrytis cinerea* (bean, tomato, and apple) [44]. In this study, the relative expression levels of two-component-system *HK* genes were analyzed under cold stress conditions with three different normalization strategies. The results showed that *HK* genes were significantly up-regulated after cold stress using stable RG(s) in normalization. However, an overestimated relative expression level in the *HK* genes transcription was obtained from qRT-PCR using *PGK* in normalization (Fig 4). The relative expression level of target gene is overestimated due to use unstable gene normalization is also found in other studies [45, 46]. These results indicated that using unstable RG(s) for qRT-PCR normalization will lead to inconsistent results. Moreover, our results suggested that the up-regulated *HK* genes participated in response to cold stress of *C*. *militaris*. However, whether or how these *HK* genes involved in cold-induced fruiting body formation of *C*. *militaris* needs further study.

## Conclusions

We systematic validated 12 candidate RGs under cold incubation during short and long cold periods, respectively, in *C*. *militaris*. Our results showed that two RGs *UBC*/*PP2A* and three RGs *UBQ*/*TUB/CYP* were identified as available for qRT-PCR normalization under cold incubation during short and long cold periods, respectively. In addition, our results indicate that failure to statistically validate RG(s) leads to inconsistent results. Moreover, the role of up-regulated *HK* genes in cold-induced fruiting body formation in *C*. *militaris* needs further study. All in all, our results provide a good starting point for accurate qRT-PCR normalization in *C*. *militaris* under cold stress and better support for understanding the mechanism of response to cold stress and fruiting body formation in *C*. *militaris* and other mushroom-forming fungi in future research.

## Supporting information

S1 Table Details on primers of two-component-system histidine kinas used for qRT-PCR analysis. (DOC)
