# Supplementary material for: Analysis of reference genes stability and histidine kinase expression under cold stress in Cordyceps militaris
Source: PLoS One. 2020 Aug 12;15(8):e0236898. doi: 10.1371/journal.pone.0236898 (PMC7423124; doi:10.1371/journal.pone.0236898)
Supplement: S1 Table — (DOC) [file pone.0236898.s001.doc]

**Table S1**: Details on primers of two-component-system histidine kinas used for RT-qPCR analysis.

| **Gene** | **Annotation** | **Accession No.** | **Primer Sequences (5’-3’)** | **Amplification Size (bp)** |
| --- | --- | --- | --- | --- |
| *CmHK1* | sensor histidine kinase | XM_006669609.1 | F: AATACGTCACCAACCGATAA R: CGCCAAAACAGAAGTAAGTC | 198 |
| *CmHK2* | sensor histidine kinase | XM_006673018.1 | F: GAGGTGACGGAGCTTACA  R: CCATGATACCATTGAGAGGC | 202 |
| *CmHK3* | autoinducer 2 sensor kinase/phosphatase luxQ | XM_006674338.1 | F: CCTTGCGGCTTACAAATAG  R: TGATGATGCTGCGATACAC | 211 |
| *CmHK4* | two-component sensor protein histidine protein kinase | XM_006665421.1 | F: GAAGCCGAAGTGTTGAGATA  R: ATTTTGTCCAAGTCGTAGTG | 197 |
| *CmHK5* | two-component osmosensing histidine kinase | XM_006666710.1 | F: CCGAAGGTTTACTTGGTG  R: GATGGTTTGCTGGAGTTG | 218 |
| *CmHK6* | histidine kinase (ethylene receptor） | XM_006673019.1 | F: CTGCGTCATTGCGTGTT  R: GCGAAAGGATGGGATAAA | 229 |
| *CmHK7* | histidine kinase (ethylene receptor) | XM_006670556.1 | F: ACCCAATCTCACCTCCCTAT  R: CATACTCGCAGGACGGAA | 190 |
| *CmHK8* | sensor histidine kinase/response regulator TcsB/Sln1 | XM_006673952.1 | F: AACACCACTAAACGGTATCA  R: GTCGCTCCAGGCTAACTT | 179 |
| *CmHK9* | sensor kinase/phosphatase luxQ | XM_006673301.1 | F: CAAGATGCCATTATCGAAGT  R: AGTGAAGGTGAGGGTGAAGTA | 165 |
